# Check-All-That-Apply (CATA) Test to Investigate the Consumers’ Perception of Olive Oil Sensory Properties: Effect of Storage Time and Packaging Material

**DOI:** 10.3390/foods10071551

**Published:** 2021-07-05

**Authors:** Maria Piochi, Giorgia Cabrino, Luisa Torri

**Affiliations:** Sensory and Consumer Science, University of Gastronomic Sciences, 12042 Pollenzo, Italy; m.piochi@unisg.it (M.P.); g.cabrino@unisg.it (G.C.)

**Keywords:** consumer acceptability, glass, polyethylene terephthalate, rapid method, sensory properties, tinplate, vegetable oils

## Abstract

Sensory changes during shelf-life of oils have been mostly studied by descriptive methods, while consumer-based approaches have been poorly explored. This study assessed the variations in consumers’ liking and sensory perception of extra virgin olive oil (EVOO) and olive oil (OO) packaged in glass, polyethylene terephthalate and tinplate. After 2, 10 and 19 months of storage, oil perception was investigated with consumers (n = 50) performing both a liking test and a check-all-that-apply (CATA) test. No significant effect of the packaging material on consumers’ response was found, whereas storage time negatively affected the sensory properties of and acceptability of OOs and EVOOs from the 10th month of storage. The CATA test results revealed the sensory changes in oils over 19 months, mainly described as a decrease in pungency for EVOO and a decrease in herbaceous and ripe fruitiness in OO. The CATA technique combined with the liking test allowed the drivers of liking (“olive” for OO and “green fruitiness” for EVOO) and disliking (“bitter” and “pungent” for EVOO) to be identified. In conclusion, the sensory approach based both on CATA technique and liking test seems promising as a rapid tool to evaluate the changes in sensory properties perceivable during the shelf-life of oils.

## 1. Introduction

Extra virgin olive oil (EVOO) is defined as “the superior category olive oil obtained directly from olives and solely by mechanical means” while olive oil composed of refined olive oils and virgin olive oils is defined as “oil comprising exclusively olive oils that have undergone refining and oils obtained directly from olives” (OO) [1]. The quality of EVOO is affected by several factors during storage, including light [2,3,4], oxygen [3,5,6] and temperature [7,8]. Furthermore, factors endogenous in oil affects the quality changes during the shelf-life, such as the initial amount of phenols: if high, it allows a low head space accumulation of off-flavor volatile substances and the best retention of sensory properties and health benefits [5]. Quality changes in EVOO during the shelf-life are essentially linked to the modifications in the initial anti-oxidant fraction and fatty acid composition [9] with oxidation constituting the major factor for the quality deterioration of olive oil [10].

The choice of the proper packaging material is crucial for the maintenance of the quality over time of oils from olives [11,12]. In fact, proper oil storage preserves the color and flavor properties of the oil including the aroma, taste and the somatosensory properties such as pungency [13]. Several packaging materials have been studied for oils from olives, including glass, metals (tin-coated steel) and, more recently, plastics [12]. The literature provides numerous comparisons between different materials [6,7,10,14,15,16,17]. Glass is among the most commonly used material for oil packaging [8,10], as it is considered the first choice as an oil container because it is impermeable to water vapor and volatile organic compounds, and it can be given light-filtering properties [14]. Metallic materials offer the advantage of total protection against light, oxygen and vapors but, since metal containers can initiate oxidative degradative reactions, current metallic material is tinplate or tin-free steel based on chromium instead of aluminum or aluminum alloys. The most common metal container is tinplate [8]. Plastic materials have been increasingly studied. Polyethylene terephthalate (PET) is the most commonly used, with many advantages including clarity, chemical inertness, low oxygen and water permeability, and excellent mechanical properties [12]. PET was studied in different versions, including colored PET (clear, green, orange, white and blue) [4] or innovative plastic film added with UV-blocker has been recently tested [6,14]. From the environmental point of view, despite being mistakenly perceived as the ones with the highest carbon footprint if considering only the end-of-life perspective, plastic materials have some environmental advantages over other materials regarding the consumption of natural resources and emissions [18], and they are recyclable [14] as for glass and tinplate.

Despite the wide literature focusing on the materials’ performance, there is currently not a univocal agreement on which material performs best, which is also due to the innovative introduced variants in classical materials. Nevertheless, it is known that an incorrect oil storage may compromise the sensorial properties of the oils by providing sensorial defects (negative perceptions) such as rancid flavor [7]. Since the decay of the sensory attributes can be detrimental for consumers’ acceptability towards the product, it is important to evaluate the sensory properties of oils during storage in order to avoid sensory defects that compromise the product quality and the acceptability by consumers. Apart from the official methods used for the commercial classification of oils into the three classes of virgin oils [19,20], typically the sensory properties of oils were described though conventional descriptive methods requiring a trained panel, such as descriptive analysis or temporal dominance of sensations [7,9,21,22]. Classical descriptive methods have the limitation of being time-consuming, due to the panel training. Moreover, the sensory evaluation of a trained assessor does not necessarily match with the perception of consumers [23] or consumers may not necessarily recognize that positive sensory attributes are related to the presence of healthy substances (e.g., polyphenols) [24]. For example, the perception of bitterness or pungency, which are considered positive attributes by trained assessors, may be instead disliked by consumers [25,26,27], especially among those not familiar with the sensory properties of EVOO of excellent quality [28].

Classical approaches used with consumers for oils envisage the evaluation of liking and the creation of preference maps [27] or the evaluation of elements of the packaging that mostly attract consumers [29,30]. Regarding rapid sensory methods, a modified version of a sorting task based on visual assessments of branded oil bottles was used with consumers to evaluate the opinions of consumers towards different oils [31]. Check-all-that-apply (CATA) was recently used with the aim of investigating oil acceptance, purchase intention, and emotional responses [26]. However, to the best of authors’ knowledge, no studies using rapid sensory techniques have been conducted yet with consumers to explore how sensory properties change during the shelf-life. Thus, the general aim of the present study was to explore the consumers’ response towards oils from olives (EVOO and OO) over the storage time by means the CATA test. The specific aims of this research were: (1) to investigate the effect of storage time on consumers’ description of the sensory attributes of oils; (2) to compare the influence of three packaging materials (green glass, GG; tinplate, TP; polyethylene terephthalate, PET) on sensory changes of oils; (3) to evaluate the impact of the sensory changes on the consumers’ acceptability for the oils over storage time.

## 2. Materials and Methods

### 2.1. Oil Samples

Two commercial categories of oil from olives (OO and EVOO) were used. The chemical parameters of the EVOO were as follows: free acidity (% oleic acid per 100 g of oil): 0.25; peroxide value (meq O_2_ Kg^−1^): 6.1; K232: 2.246; K270: 0.176; ΔK: −0.001; total polyphenol content: 300 ppm. EVOO and OO were packed on the same day (January 2019) in 1 L containers made of three different materials: green glass (GG), tinplate (TP), clear polyethylene terephthalate (PET). The sealed oil samples were provided to the Sensory Laboratory of the University of Gastronomic Sciences (Pollenzo, Italy), where they were stored for 19 months in the dark in an air-conditioned room at 22.0 ± 0.5 °C and a relative humidity of 55%. Oil packages were randomly sampled for sensory evaluation after 2, 10 and 19 months of storage (T2, March 2019; T10, January 2020; T19, October 2021). At each control time, nine oil samples were analyzed for each oil category (OO, EVOO) from all possible combinations of packaging material and storage time (T2_GG; T2_TP; T2_PET; T10_GG; T10_TP; T10_PET; T19_GG; T19_TP; T19_PET). At each session of sensory evaluation, a new bottle of oil was opened and used. For the sensory tests, the oil samples (15 mL) were presented at room temperature (20 ± 1 °C), in disposable clear polylactic acid (PLA) cups (96 mL) codified with three-digit random codes and covered with aluminum foil. We opted for single use containers that allowed us to see the color of the oil samples, since containers different from the official glass for olive oil tasting [32] have been previously used in test with consumers [28]. Oils were poured into the PLA container approximately 30 min prior each evaluation.

### 2.2. Subjects

Three groups of 50 adult subjects (68% non-smokers; 74% Italians; 61% females; age range: 18–52 years, mean age: 23 years) joined three tasting sessions, each corresponding to a control time (T2, T10, T19). The sample size was chosen in agreement with Meilgaard, Civille and Carr [33] who recommend a minimum of 50 subjects for affective methods. Although the subjects were partially different at various control times, all participants came from the same educational environment, as they were recruited among students and staff of the University of Gastronomic Sciences (Pollenzo, Italy), without any health conditions affecting their taste and smell ability. Most participants were regular consumers of EVOO (34% consumed it more than once a day; 41% consumed it daily; 17% consumed it more times per week; 5% at least once per week; 2% from 1 to 3 times per month and only 1% consumed it less than once a month) and occasional consumers of OO (13% consumed it more than once per day; 26% every day; 17% several times per week; 5% at least once per week; 9% from 1 to 3 times per month and 31% consumed it less than once a month). The whole study complies with the Declaration of Helsinki for Medical Research involving Human Subjects and was approved by the Ethics Committee of the University of Gastronomic Sciences (Ethics Committee Proceeding n. 2019.02). Prior to the test, participants were informed about the general aim of the study and the evaluation procedure and signed an informed consent form. Participants were asked to not eat/drink/smoke or wear perfume for at least one hour before the evaluation session.

### 2.3. Evaluation Procedure

At every session, participants answered a short questionnaire, and then performed a liking test and a CATA question test to evaluate the perception associated with each oil. For the tasting, all participants assessed the three samples of OO as the first set (PET, GG, TP) and the three samples of EVOO as the second set (PET, GG, TP). Samples of OO and EVOO were served in separated trays. The order of samples was randomized and balanced among subjects within each set.

Firstly, subjects were asked to provide socio-demographic data (gender, age, nationality), to declare their smoking status (non-smoker, smoker, ex-smoker status), their frequency of consumption of EVOO and OO separately (less than once a month; one or three times a month; at least once a week; several times a week; every day; more than once a day). Secondly, for each sample, participants rated their liking for the appearance, taste, flavor, mouthfeel and overall liking on a 9-point hedonic scale (1 = dislike extremely, 9 = like extremely; [34]). Thirdly, participants were asked to select from a list of sensory attributes all the descriptors that they perceived in each sample. The list of attributes was presented in a randomized, subject-balanced order and included 21 descriptors for OO and 16 descriptors for EVOO (Table 1). CATA attributes were selected based on literature (including defects) and on terms elicited by consumers in a pre-test, since it is known that terms for CATA can be predefined by a trained panel or consumers [35]. Data were collected with the software Fizz version 2.47 B (Biosystèmes, Couternon, France) in computerized individual booths under white light. The choice of conducting the evaluation under white light (and thus enabling the visualization of the appearance and color) was driven by the will for reproducing a tasting condition close to what is normally experienced by consumers in a real context of consumption (that includes the appearance). In fact, even if the appearance is not considered a quality parameter according to the official panel test method [19] and the acceptability for oil color greatly varies across consumers with different familiarity and commitment for oil [28], the color of oil is definitely one of the major intrinsic factors evaluated by consumers impacting the oil choice [36]. Participants took approximately 10–15 min to conclude each evaluation session.

### 2.4. Data Analysis

Data regarding OO and EVOO were always separately analyzed. All data are expressed as mean values and standard errors in the text.

Liking data obtained at the different control times were separately submitted to three-way ANOVA models (random factor: subjects; fixed factors: material and storage time; two-way interaction model: material × storage time) to investigate the effect of material, time, and material over time on liking of the different sensory modalities evaluated separately (appearance, taste, flavor, mouthfeel and overall liking). All ANOVA models were followed by Tukey’s HSD Test (*p* ≤ 0.05) to test for significant differences between mean values. Based on the frequency of consumption of OO and EVOO, consumers were divided in three groups: those having a higher frequency of EVOO consumption, those consuming more OO than EVOO and those declaring an equal frequency of consumption of OO and EVOO. This classification was used to verify if the frequency of consumption of oil could affect the acceptability for different oil category with a one-way ANOVA (fixed factor: group of consumers with different frequency of consumption).

For the CATA test data, the number of times each descriptor was chosen by the consumers for each product was calculated to obtain an occurrence matrix. Cochran’s tests were conducted on the occurrences matrices to test the significance of the attributes in discriminating among the nine samples (three control times × three packaging materials) of each oil category. A correspondence analysis (CA) was conducted on the matrix of the resulting significant attributes to obtain a perceptual map showing the sensorial description of samples. Perceptual maps must be interpreted based on a criterion of spatial proximity between both attributes and samples (i.e., samples that were spatially close to each other were found to be perceptually similar, and samples close to an attribute reported high percentages of choice of that attribute; conversely, samples distant in the two-dimensional space were perceived as different overall, and samples spatially distant from an attribute were characterized by low frequencies of choice of that attribute). A penalty analysis (PA) was conducted between the significant attributes (occurrences) and the overall liking to find the positive and negative drivers of liking for the samples. The PA provided two graphs (for OO and EVOO, respectively) depicting on the *Y*-axis the mean impact of the attributes on liking (whether positive or negative) and on the *X*-axis the percentage of subjects choosing that attribute across the samples. A vertical dotted line on the *X*-axis depicts the threshold over which the results are considered significant (20%). A k-means cluster analysis (clustering criterion: Trace (W); number of groups: three) was conducted on the occurrence matrix of the significant attributes of samples to find clusters of OO and EVOO samples based on their sensorial similarity. All analyses were conducted with XLStat 2020.3.1 (Addinsoft, Boston, MA, USA).

## 3. Results

### 3.1. Effect of Storage Time and Packaging Material on Acceptability of Oils

The three-way ANOVA model (random factor: subjects; fixed factors: material and storage time; two-way interaction model: material × storage time) revealed a significant effect of the storage time on liking. Storage time negatively affected liking of all sensory modalities in both oil categories, OO and EVOO (Table 2). As expected, fresh oils (T2) were significantly more liked than oils at the end of their shelf-life (T19). A significant (*p* < 0.01) decrease of liking for most sensory modalities occurred after 10 months. For both oil categories at the end of the shelf-life, oils remained acceptable, especially EVOO, which received higher scores than OO, in general.

The factor “material” and the interaction “material × storage time” did not significantly (*p* > 0.05) affect the acceptability for both OO and EVOO for any of the considered sensory modalities (appearance, odor, taste, flavor mouthfeel, overall liking), suggesting that the different materials had comparable performances in terms of acceptability.

Based on the segmentation conducted on consumers’ oil frequency of consumption, the ANOVA results showed that the frequency of consumption significantly (*p* < 0.05) affected the liking of all sensory modalities for both oil categories. Those consumers equally familiar with both oil classes had the highest acceptability for both oil classes compared to both those with a higher frequency of EVOO consumption and those consuming more OO than EVOO.

### 3.2. Effect of Storage Time and Packaging Material on Sensory Description of Oils

The results from the Cochran’s Q test revealed that 16 attributes (“artichoke”, “buttery”, “chemical”, “fruity”, “fusty/muddy sediment”, “herbaceous”, “metallic”, “musty/stale”, “nutty”, “olive”, “oxidized”, “rancid”, “soapy”, “sweet”, “tomato leaf”, “vinegary”) and 11 attributes (“astringent”, “buttery”, “fusty/muddy sediment”, “mineral”, “nutty”, “pungent”, “rancid”, “ripe fruity”, “soapy”, “vinegary”, “woody”) included in the CATA test were significant (*p* < 0.05) in discriminating among the nine samples differentiated for packaging material and storage time, respectively, for OO and EVOO. Therefore, the results suggest that consumers perceived large differences in the sensory properties of the evaluated oils during the sample aging.

**Figure 1:**
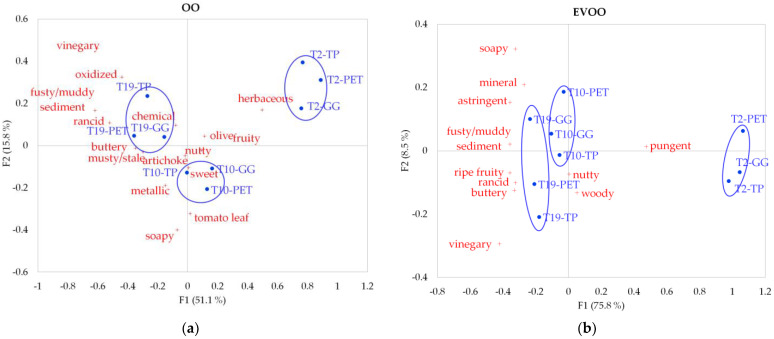
Representation of the olive oil samples and the terms in the first and second dimensions of the correspondence analysis of the CATA counts as a function of storage time (2, 10 and 19 months) and packaging material (Polyethylene terephthalate, PET; Green glass, GG; Tinplate, TP). (**a**) Olive oils; (**b**) Extra-virgin olive oils. Circles in the figures represent homogenous groups obtained from k-means cluster analysis.

From the CA performed on the significant attributes from CATA questionnaire, considering all the control times and the three materials, two perceptual maps were obtained for OO and EVO, respectively. The sensory map of OO (Figure 1a) reported 66.9% of the total inertia. The first dimension showed a clear discrimination of oils as a function of the storage time, with the freshest samples (T2) positioned on the right part of the graphs and the oils at the end of their shelf-life (T19) positioned on the left quarters of maps. The clear discrimination of the oil samples as a function of the storage time was confirmed both for OO and EVOO by the results of the k-means cluster analysis (Figure 1). T2 samples were described by high occurrences of positive sensorial attributes, such as “herbaceous” and “fruity” for OO. On the contrary, the T19 samples were mostly associated with negative sensations linked to sensorial defects, such as “vinegary”, “oxidized”, “fusty/muddy sediment”, “rancid”, “musty/stale”, and “buttery” for OO. Samples stored for 10 months presented intermediate sensory properties. A similar distribution of the samples was observed on the sensory map of EVOO, depicting 84.3% of the total inertia for EVOO (Figure 1b). However, a less clear discrimination among oils stored for 10 and 19 months was noticed. Oils stored for 2 months were mainly perceived as “pungent”, as opposed to the samples stored longer (T10 and T19), which were described as more “ripe fruity” and with negative attributes (“fusty/muddy sediment”, “rancid”, “buttery”, “soapy”, “vinegary”).

In both graphs, samples analyzed at the same control time but packaged in different material containers were remarkably close to each other, suggesting minimal differences in terms of sensory changes during storage. Thus, the effect of time was more pronounced than the effect of the material in preserving the sensory quality of oils over storage.

### 3.3. Impact of the Sensory Attribute Perception on Liking

**Figure 2:**
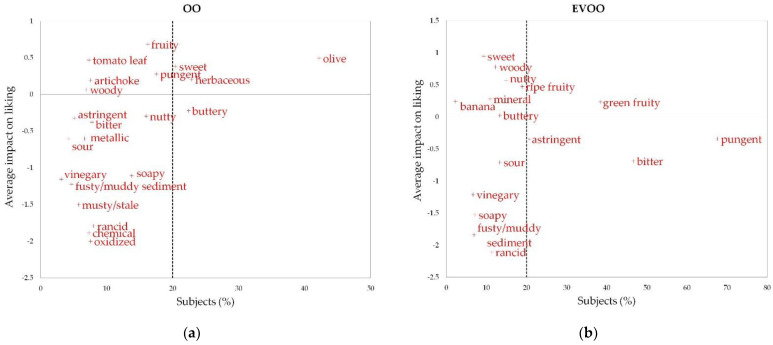
Penalty charts showing the impact of the sensory attribute perception on the overall liking of oils estimated with consumers. (**a**) Olive oils; (**b**) extra-virgin olive oils.

The results of the penalty analysis conducted to investigate the relationship between the overall liking data and the attribute occurrences obtained for OO and EVOO are reported in Figure 2a,b, respectively, showing the mean drops in overall liking as a function of the proportion of consumers that checked an attribute across all samples. The influence of each attribute on liking increased if the attribute had a high percentage of choices and a high mean impact (attribute positioned in the upper right quadrant). Instead, if an attribute had a negative mean impact value, its presence in the sample (perception of that sensation) was negative for the liking. Based on these criteria, for OOs the flavors of “olive”, “herbaceous” and “sweet” were found to be positive drivers of liking, whereas “buttery” had a slight negative impact. For EVOO, the “green fruity” attribute was found to have a positive effect on liking. On the contrary, “pungency” and “bitterness”, despite being considered positive attributes by the official Panel test method [19], were found drivers of disliking among participants in the present study, as for astringency.

The sensations linked to defects (for OO: “oxidized”, “chemical”, “rancid”, “musty/stale”, “fusty/muddy sediment”, “vinegary”, “soapy”, etc.; for EVOO: “soapy”, “fusty/muddy sediment”, etc.) had a general negative impact both on OO and EVOO liking, despite being chosen by a proportion of subjects lower than 20%, which is considered the cut-off for reliable results of the penalty analysis [37].

## 4. Discussion

### 4.1. Effect of the Storage Time and Packaging Material on Acceptability

According to [38], “shelf-life” can be defined as a finite length of time after production and packaging during which the food product retains a required level of quality under well-defined storage condition. It is known that oil should be stored for as short a time as possible to preserve its quality, with the general assumption that it should not exceed the production year of storage [12]. EVOO shelf-life has been assessed to be even longer, at 12–18 months [3]. In the current study, we explored the impact of the storage time and of the packaging material on the acceptability and sensory properties perceived by consumers for OO and EVOO. Results showed that liking slightly decreased with aging of oil in both product categories (OO, EVOO) over 19 months at the considered storage conditions. This outcome was expected, as previous literature showed a significant decrease in both the sensory evaluation and quality parameters over 12 months in EVOO [7]. The temperature of storage that we used (22 °C) was chosen as representative of room temperature [6]. The decrease in liking that we observed at this temperature was less evident compared to the decreased sensory performance found by other authors [7] who stored the samples at 37 °C, probably because a higher temperature (representative of an elevated ambient temperature encountered during the summer) accelerated the degradation kinetics in oils, inducing a more pronounced sensorial decrease. In our case, the main decrease was observed after 10 months of storage, but the oils remained acceptable even at the 19th month of storage. This fact represents an important confirmation for the industries operating in the extra virgin olive oil and olive oil production/bottling which intend to keep on the market their products up to 18 months, in agreement with the Italian Law 9/2013 that establishes this duration as the minimum term of conservation of oils from olive (“best before…”) [39].

The current study provides the first evidence regarding consumers’ liking of oils contained in different packaging materials during storage time. Results did not highlight an evident effect of the packaging material on the acceptability of oils, meaning that at the considered preservation conditions (22 °C, relative humidity 55%) oils packaged in PET, GG and TP were equally liked among consumers. Some minor sensorial differences were found (discussed below). However, the sensorial variations were probably not enough to significantly discriminate the samples in terms of liking. The fact that the liking of the appearance did not significantly decrease was in agreement with a previous study showing that the olive oil color was unaffected by packaging materials with different oxygen transmission rates over 12 months of storage time [6]. Considering different bottling materials, PET bottles in Italy normally cost less than green glass or tinplate. Since the packaging seems not to strongly affect the sensory performance in terms of diversification, one could argue that producers may easily opt for the cheapest version of material. However, in the effort of moving towards sustainable food productions, a careful evaluation of the sustainability issues of the material is suited [40], since this aspect is both necessary for the environment and increasingly cared about by consumers [41].

As mentioned in the introduction, several studies instrumentally assessed the impact of the packaging and storage time on the oil quality described by relevant indices (acidity, peroxide value, K232 and K270, total phenolic content). Traditional studies on oil storage suggested that oil stored in stainless steel had better qualitative level (with significantly higher levels of phenols and lower values of the oxidative indices) than that stored in transparent clear and green colored glass [16] and that stainless and dark glass appeared to be the most adequate packaging materials compared to clear PET or clear glass [42]. However, recent studies suggest other innovative packaging materials with outstanding oxygen barriers (e.g., flexible PET with UV-blocker) can outperform glass in terms of quality parameters [14], suggesting the need to further explore the impact of new innovative solutions.

### 4.2. Effect of the Storage Time and Packaging Material on Sensory Description of Oils

As expected, the impact of storage time on the sensory properties of oils was found to be more relevant than the impact of the material. The fact that fresh oils (T2) were described with positive attributes (e.g., “herbaceous” for OO or “pungent” for EVOO) while the samples stored for 19 months were characterized by negative attributes (e.g., “oxidized”, “chemical”, “rancid” and “reheated/stained” for OO), indicates an obvious negative effect of time on the state of storage of oils with an increase in the perception of sensory defects by consumers. Several deterioration reactions are known to occur during oil storage, out of which the most common variation is generally recognized with the term “rancidity”, including hydrolytic and oxidative rancidity [12]. Thus, the increase in occurrences of terms such as “rancid” detected by consumers with aging is in agreement with the literature. Moreover, it is well known that the total phenols compounds found by HPLC-UV-MS significantly decreased during the storage [43]. Since the total phenols content is positively and strongly correlated with “bitterness” and “pungency” [44], in this sense, the decrease of “pungency” in EVOO that was observed in the current study is in line with findings of literature indicating a soften of oils during storage.

“Bitterness” and “pungency” have been widely studied in oils. Typically, the intensity of these two descriptors decrease with storage time in oils [45], as well as the sensations of “green fruitiness” [7]. In the current study, both descriptors were found to be negative drivers of liking for EVOO, despite being considered positive attributes by the official panel test method [19]. However, the fact that bitter taste was a negative driver of liking in EVOO among consumers fully confirmed several previous studies demonstrating that this sensation is generally disliked in oils [25,26,27]. A recent review concluded that the sensory perception of the consumers regarding a high-quality olive oil, typically being characterized by both bitterness and pungency at different intensities, matches to the perception of panel tasters in countries with a tradition of virgin olive oils consumption, but this correlation was not observed with consumers in non-traditional olive-oil-consuming countries [46]. Although most of our consumers declared to be regular EVOO consumers, many of them were living in the Piedmont region (which is not historically among the regions in Italy that consume the most oils compared to other regions, for example in the south) and 26% of participants were not Italian, thus probably without a strong tradition of olive oils consumption. This characteristic of the participants could partially explain why those two attributes were not appreciated [25]. However, the fact that the “green fruity” in EVOO was found as a positive driver of acceptability was in agreement with previous studies indicating the importance of fruitiness for consumers (both green and ripe) [28]. For both OO and EVOO, the fact that “sweet” was a positive descriptor for liking in opposition to “bitter” is in agreement with the fact that those two sensations are often found negatively correlated from a sensorial point of view [47]. The fact that some negative descriptors (identified as defects) were associated with some oil samples, especially with those stored longer, is not surprising. In fact, important qualitative differences have been previously found among the oils belonging to the same commercial class of “extra virgin olive oil”, with oils distinguishing themselves in terms of both chemical parameters and sensory properties [48]. However, the occurrences of negative descriptors (some of which identified defects) remained quite low even at the highest storage time (T19).

From a methodological point of view, the current study adopted the check-all-that-apply test as rapid technique to obtain the consumers’ sensory description of the oils. The CATA test has been previously applied to link consumers’ emotions to the sensory characterization of a wide range of different food products (salted snacks, potato chips, yoghurt, cheese, snack bars and fruit, nuts, chocolate, fruit, and processed tomato products) [49,50]. Regarding oils, CATA was recently applied to investigate the sensory characterization of commercial and organic extra virgin olive oils [26] and to evaluate the acceptability and sensory characteristics of five sesame oil samples prepared with different technological processes [51]. To the best or our knowledge, the current study is the first to apply the CATA test to evaluate consumers’ perception of the sensory properties of food products over time. Since consumers were able to detect the negative sensations in oils that are associated with oil aging, the obtained results showed that the CATA technique could be highly informative to evaluate the changes in sensory properties of during the shelf-life.

## 5. Conclusions

No significant effects of the packaging material (PET, GG, TP) on consumers’ liking nor on the sensory properties of the samples were found at the considered storage conditions (22 °C, 55% of relative humidity, in the dark, for 19 months). On the contrary, storage time negatively affected the sensory properties and acceptability of OO and EVOO samples from the 10th month of storage. The CATA test performed with consumers was effective in revealing the sensory changes in oils over 19 months, mainly described as a decrease in “pungency” for EVOO and a decrease in “herbaceous” properties and “ripe fruitiness” in OO. Moreover, the combination of the CATA technique with the liking test allowed the drivers of liking (“olive” flavor for OO and “green fruitiness” for EVOO) and disliking (“bitter” and “pungent” for EVOO) to be identified. In conclusion, a sensory approach based both on CATA technique and liking test seems promising as a rapid tool to evaluate the changes in sensory properties perceivable during the shelf-life of commercial oils.

## Figures and Tables

**Table 1:** Attributes used for the CATA test for olive oil (OO) and extra virgin olive oil (EVOO).

Olive Oil (n = 21)	Extra Virgin Olive Oil (n = 16)
Artichoke	Astringent
Astringent	Banana
Bitter	Bitter
Buttery	Buttery
Chemical	Fusty/Muddy sediment
Fruity	Green fruity
Fusty/Muddy sediment	Mineral
Herbaceous	Nutty
Metallic	Pungent
Musty/Stale	Rancid
Nutty	Ripe fruity
Olive	Soapy
Oxidized	Sour
Pungent	Sweet
Rancid	Vinegary
Soapy	Woody
Sour	
Sweet	
Tomato leaf	
Vinegary	
Woody	

**Table 2:** Effect of storage time on the acceptability of OO and EVOO (T: storage time; F: Fisher‘s ratio).

Oil	Sensory Modality	Storage Time				
		T2	T10	T19	F	*p*-Value
OO	Appearance	6.0 ± 0.1 a	5.1 ± 0.1 b	5.4 ± 0.1 b	16.0	<0.0001
	Odor	5.9 ± 0.1 a	5.2 ± 0.1 b	5.1 ± 0.1 b	13.8	0.000
	Taste	5.7 ± 0.1 a	5.0 ± 0.1 b	5.2 ± 0.1 b	7.4	0.001
	Flavor	5.7 ± 0.1 a	5.1 ± 0.1 b	5.1 ± 0.1 b	5.9	0.003
	Mouthfeel	5.7 ± 0.1 a	4.9 ± 0.1 b	5.1 ± 0.1 b	11.0	0.000
	Overall liking	5.8 ± 0.1 a	4.9 ± 0.1 b	5.1 ± 0.1 b	12.0	0.000
EVOO	Appearance	7.7 ± 0.1 a	7.3 ± 0.1 b	7.2 ± 0.1 b	6.9	0.001
	Odor	7.4 ± 0.1 a	6.7 ± 0.1 b	6.4 ± 0.1 b	21.0	<0.0001
	Taste	6.6 ± 0.1 a	6.2 ± 0.1 ab	5.8 ± 0.1 b	6.9	0.001
	Flavor	6.6 ± 0.1 a	6.1 ± 0.1 b	5.7 ± 0.1 b	7.0	0.001
	Mouthfeel	6.7 ± 0.1 a	5.9 ± 0.1 b	5.8 ± 0.1 b	10.8	0.000
	Overall liking	6.7 ± 0.1 a	6.1 ± 0.1 b	6.0 ± 0.1 b	6.9	0.001

Different letters within rows indicate significant mean values (Tukey’s HSD Test, *p* < 0.05).

## Data Availability

Restrictions apply to the availability of these data. Data were obtained under a private agreement with Consorzio Nazionale Riciclo e Recupero Imballaggi Acciaio and are available from the author with the permission of Consorzio Nazionale Riciclo e Recupero Imballaggi Acciaio.
